# Biomimetic nanotherapeutics for targeted drug delivery to glioblastoma multiforme

**DOI:** 10.1002/btm2.10483

**Published:** 2023-02-14

**Authors:** Xin Yuan Lim, Sharah Mae Capinpin, Nagarjun Bolem, Aaron Song Chuan Foo, Wai‐Cheong George Yip, Alan Prem Kumar, Daniel Boon Loong Teh

**Affiliations:** ^1^ MBBS Programme Yong Loo Lin School of Medicine, National University of Singapore Singapore Singapore; ^2^ Department of Pharmacology Yong Loo Lin School of Medicine, National University of Singapore Singapore Singapore; ^3^ NUS Centre for Cancer Research Yong Loo Lin School of Medicine, National University of Singapore Singapore Singapore; ^4^ Department of Surgery, Division of Neurosurgery National University Hospital Singapore Singapore; ^5^ Department of Anatomy Yong Loo Lin School of Medicine, National University of Singapore Singapore Singapore; ^6^ Department of Biochemistry Yong Loo Lin School of Medicine, National University of Singapore Singapore Singapore; ^7^ Department of Ophthalmology Yong Loo Lin School of Medicine, National University of Singapore Singapore Singapore; ^8^ Neurobiology Life Science Institute, National University of Singapore Singapore Singapore

**Keywords:** biomimetic, glioblastoma, nanoparticle, nanotechnology

## Abstract

Glioblastoma multiforme (GBM) is an aggressive brain tumor with poor prognosis and high mortality, with no curative treatment to date as limited trafficking across the blood–brain barrier (BBB) combined with tumor heterogeneity often leads to therapeutic failure. Although modern medicine poses a wide range of drugs that are otherwise efficacious in treating other tumors, they often do not achieve therapeutic concentrations in the brain, hence driving the need for more effective drug delivery strategies. Nanotechnology, an interdisciplinary field, has been gaining immense popularity in recent years for remarkable advancements such as nanoparticle (NP) drug carriers, which possess extraordinary versatility in modifying surface coatings to home in on target cells, including those beyond the BBB. In this review, we will be highlighting recent developments in biomimetic NPs in GBM therapy and how these allowed us to overcome the physiological and anatomical challenges that have long plagued GBM treatment.

## 1 INTRODUCTION

Glioblastoma multiforme (GBM) is the most prevalent and aggressive brain malignancy in adults, accounting for nearly half of malignant central nervous system (CNS) tumors with no curative treatment to date.^1^, ^2^, ^3^ The median overall survival (OS) of GBM patients is 15 months, with only 5.5% of patients surviving beyond 5 years from initial diagnosis.^4^ The current standard treatment follows the STUPP protocol which comprises maximal tumor tissue safe resection followed by concurrent chemo‐ and radiotherapies for 6 weeks and thereafter a 6‐month regime of oral temozolomide (TMZ).^5^ Unlike surgery elsewhere in the body where en‐bloc resection can be performed, in the brain, due consideration has to be given to balance both oncological and functional outcomes when resecting infiltrative lesions like GBM. Not uncommonly, the risk of inflicting irrevocable neurological damage precludes complete exenteration of the entire tumor during GBM surgery. In addition, while necessary for controlling tumor progression, chemoradiation in itself causes numerous undesirable side effects that are not well tolerated by most patients. Furthermore, nearly all GBM cases recur with a more aggressive form of tumor, often with a poorer prognosis and lowered median OS of 2–9 months,^6^ driving the need to develop more effective treatment strategies to improve patients' overall prognosis.

Glioblastoma multiforme is a grade IV primary brain malignancy arising from astrocytes, a type of glial cell which supports neurons in the CNS. Astrocytes are the most prevalent cells in the brain, outnumbering neurons by more than five‐fold and serving an equally vital function. Beyond providing nutrients to neurons, they play key roles in regulating the blood–brain barrier (BBB) and maintaining interstitial fluid homeostasis—a crucial factor to facilitate synaptic transmission.^7^

The BBB consists of astrocytes anchoring pericytes and extracellular matrices to the basement membrane of an endothelial cell layer that forms tight junctions (TJs), which are responsible for regulating the movement of substances across the BBB. The diffusion of small lipophilic molecules is facilitated by important transmembrane proteins within TJs, which include zona occludens (ZO), claudins, and junctional adhesion molecules.^8^ Retrograde movement of substances back into the systemic circulation is facilitated by efflux transporters present within the BBB, which include P‐glycoprotein (P‐gp), breast cancer resistance protein (BCRP), and organic anion transporting peptide (OATP).^9^ Astrocytes play a supportive, yet critical role by releasing soluble factors such as vascular endothelial growth factors (VEGFs), matrix metalloproteinases (MMPs), endothelins, angiopoietin‐1, and glial‐derived neurotrophic factor, which are essential for the maintenance and modulation of the BBB structure. These aspects allow the BBB to inhibit paracellular transport of around 98% of small molecules and nearly 100% of macromolecules.^10^ Though its protective role is vital, the BBB's highly selective nature unfortunately creates a formidable barrier impeding the delivery of drugs to the CNS.

This is not necessarily the case with GBM however, as the rerouting of vasculature to the growing tumor often destabilizes the BBB. The “leaky barrier,” or blood–brain tumor barrier (BBTB), allows macromolecules designed for targeted drug delivery to bypass the normally impregnable BBB. Unfortunately, high‐grade gliomas rapidly infiltrate surrounding tissues where the BBB is not yet compromised, making the BBTB as a sole delivery route via the enhanced permeability and retention (EPR) effect an unreliable strategy.^11^ The EPR effect refers to the ability of molecular compounds to accumulate and retain in the vascularized areas of the tumor. There is a clear need to identify ways to enhance drug delivery across the BBTB and fortunately, recent studies have shown that a solution capitalizing on this unique therapeutic avenue may be found in nanoparticles (NPs).

Nanoparticles range in size from 1 to 100 nm, and can be synthesized using inorganic, polymeric, or organic materials.^12^ Critically, they are able to carry a therapeutic payload and efficiently home in on their target cells. Multiple molecular subtypes of NPs exist, including lipid‐based, polymeric, and metallic variants.^13^ This allows flexibility in surface modification and enables them to accommodate a wide range of therapeutics, making them ideal vehicles for drug delivery. NPs such as the metallic subtypes are also synergistic with other treatment adjuncts, such as photodynamic therapy.^14^ Hence, it is no surprise that NPs have become an increasingly popular choice for drug delivery in the newly developing paradigm of GBM treatment.^15^

**FIGURE 1:**
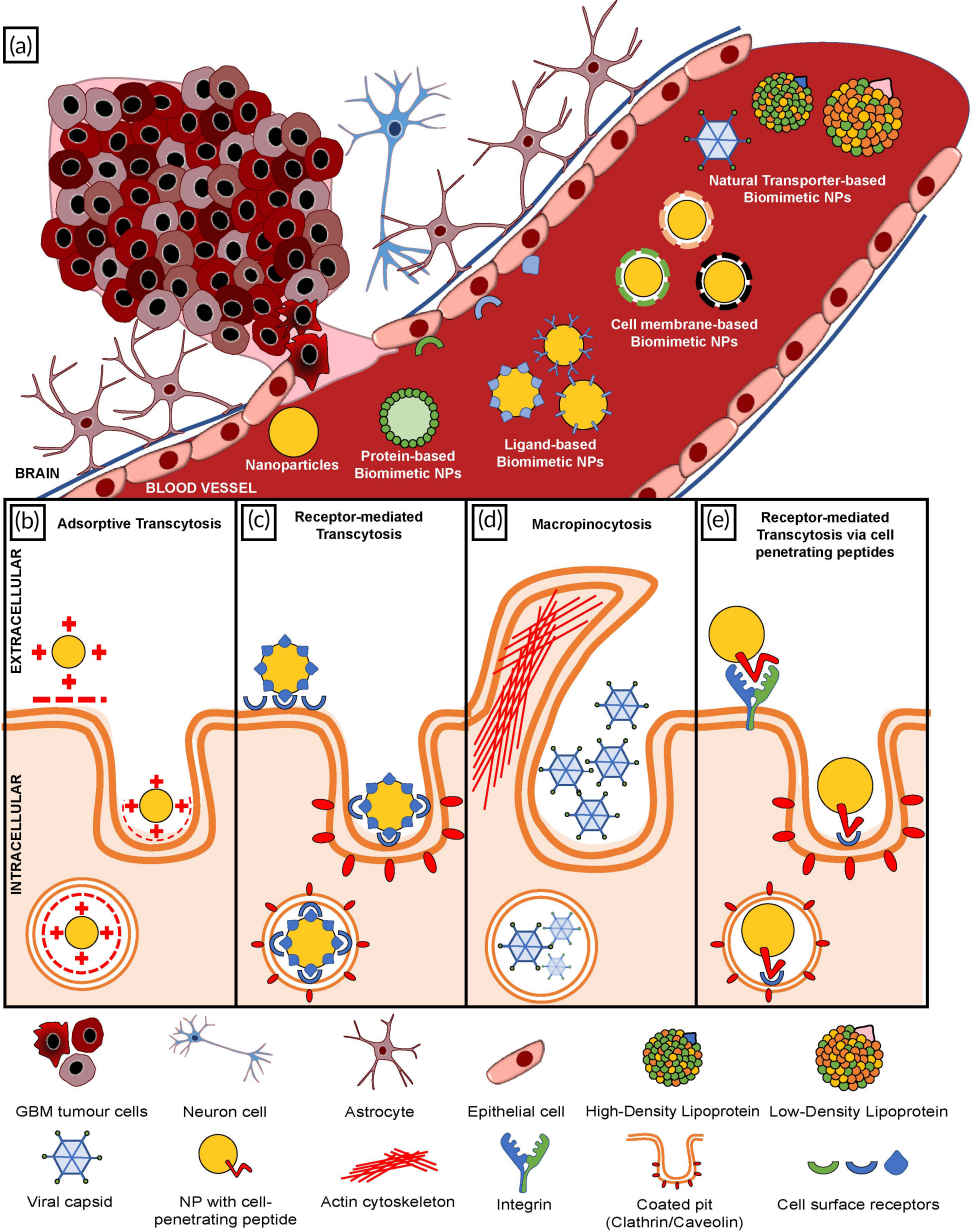
Nanoparticles (NPs) and biomimetic NPs developed for glioblastoma multiforme (GBM) therapeutics and their various drug delivery methods. (a) Different NPs and biomimetic NPs that have been successfully developed for GBM therapeutics. (b) Adsorptive transcytosis. (c) Receptor‐mediated transcytosis. (d) Macropinocytosis. (e) Receptor‐mediated transcytosis via cell‐penetrating peptides.

In this review, we discuss current GBM therapeutic strategies and their associated challenges, as well as demonstrate how NPs could be a novel approach to overcome the limitations in conventional GBM treatment. In Figure 1a, we outline the different ways NPs have been enhanced using biomaterials to increase uptake at the BBB and GBM tumor cells. Combinatorial therapeutics involving synergy between biomimetic NPs and other adjuncts will also be discussed, in hopes that we can present a novel perspective on the potential future of GBM treatment.

## 2 CURRENT PARADIGM OF GBM TREATMENT

The current standard of care relies solely on relatively conventional treatments, and patient outcomes remain largely dismal despite decades of development. Surgical cytoreduction of the tumor mass is the current cornerstone of treatment, and aims to alleviate tumor mass effects while collecting samples for immunohistochemical profiling to identify potential targetable markers.^16^ Studies show that resecting more than 98% of the tumor mass greatly improves OS,^17^ but is often unachievable as a balance must be struck to minimize damage to the remaining healthy tissue. Hence, there is a need to carefully assess GBM patients and the tumor prior to surgery to determine the extent of resection that can provide the maximum survival and functional benefit.^18^ This is followed by concurrent administration of TMZ with radiation therapy and six cycles of TMZ monotherapy thereafter.^3^

Temozolomide, a lipophilic molecule that can cross the BBB, is a monofunctional alkylating agent which methylates DNA and creates cytotoxic DNA adducts such as O6‐methylguanine (O6MG). O6MG causes the misincorporation of thymine in place of cytosine during DNA synthesis, resulting in base‐pair mismatches. The mismatch repair (MMR) system will be activated and halt DNA synthesis. If repair is unsuccessful, DNA replication fails and double‐strand breaks will lead to cytotoxic lesions and apoptosis.^19^ TMZ's mechanism of action relies on the rapid mitotic rate of malignant cells, which will have a higher rate of O6MG‐induced apoptosis. However, relatively high resistance rates to TMZ is a major challenge in GBM therapy. The primary mechanism of resistance to TMZ is through the O6MG DNA methyltransferase (MGMT) which converts O6MG back to guanine.^20^ To overcome this, several clinical trials have been implemented to evaluate drugs capable of silencing MGMT and evidence of improved patient outcomes is on the horizon. Bortezomib, an inhibitor of the 20S proteasome chymotrypsin‐like activity, has already been approved for use in the treatment of myeloma and lymphoma. Furthermore, preclinical experiments have demonstrated the ability of bortezomib in crossing the BBTB and decreasing cerebral MGMT expression, leading to sustained tumor repression, prolonged progression‐free time, and OS.^21^ A small proportion of TMZ resistance is also attributed to mutations affecting MMR expression. For TMZ to be effective, target cells must have a functioning MMR system. Although MMR genes are rarely mutated, some resistant GBM cell lines have a reduced expression of related genes such as *MSH2* and *MSH6*.^22^

In addition to TMZ resistance, GBM's angiogenic tendencies also pose a significant hurdle to treatment.^23^ Angiogenesis refers to the formation of new blood vessels which is critical for tumor growth. As any tumor mass enlarges and outgrows its blood supply, cells at the periphery become hypoxic and undergo necrosis. The production of VEGF to promote angiogenesis therefore becomes essential for tumor survival.^24^ In GBM, the abundant secretion of proangiogenic factors such as VEGF facilitates the extensive microvascular proliferation and rapid tumor growth, contributing to a more aggressive and fatal disease.^25^ Bevacizumab, an immunotherapeutic drug, binds to VEGF and prevents it from binding to its receptor. This reduces neovascularisation, normalizes vasculature, and eliminates GBM tumor cells that express VEGF.^26^, ^27^ It is most commonly used in patients experiencing GBM recurrence as phase II trials have demonstrated improvement in progression‐free survival (PFS) in this select group.^28^ Unfortunately, bevacizumab is still not part of standard GBM therapy as it has not shown to demonstrate any significant improvement in OS.^29^, ^30^ It is also not curative as tumor mutations lead to activation of alternative angiogenic pathways, or reduce reliance on angiogenesis for proliferation.^31^

Another major factor limiting the efficacy of current GBM treatment is the highly heterogenous nature of the tumor. Even within the same tumor mass, there can be multiple clonal and subclonal populations, each marked by distinct genetic alterations.^32^ Most recurrent GBM tumors also differ from the initial lesion. This limits the efficacy of single‐target therapies. Mutations can affect a variety of genes regulating critical pathways, such as MEK/ERK and PI3K/AKT/mTOR signaling channels.^33^ EGFR in particular is often overexpressed or constitutionally activated. This has led to the exploration of EGFR inhibitors such as erlotinib and gefitinib as anti‐GBM therapies, given their success in the treatment of EGFR‐positive lung cancers. However, despite their initial positive outlook, these drugs have yet to demonstrate significant improvement in OS and were instead found to cause unacceptable toxicities during GBM treatment. Their inability to reach therapeutic concentrations in the brain also contributed to their low therapeutic value.^34^ In addition, the Notch signaling pathway has been found to be associated with the establishment and maintenance of cancer stem cell niches, leading to gamma secretase inhibitors (GSIs) being assessed as potential GBM therapeutics. Unfortunately, despite being well‐tolerated in a combination therapy with TMZ and radiotherapy, GSIs showed inconsistent BBB penetration, especially at sites where the BBB remained intact.^35^ Of late, immune checkpoint inhibitors such as ipilimumab target cytotoxic T‐lymphocyte‐associated protein 4 (CTLA‐4) and have been shown to be effective in shrinking tumor and prolonging survival in preclinical studies. Clinical trials are currently taking place to determine its efficacy in GBM.^36^

Novel therapeutic strategies such as vaccine therapy using tumor peptides, tumor cells, and dendritic cells have also been gaining traction in recent years. A clinical trial targeting cytomegalovirus pp65 protein, which is found in a number of GBM tumors, has shown significant increase in PFS as well as OS.^37^

Many drugs have been explored for GBM treatment but none have yet to succeed in the clinical setting. While the above‐mentioned examples have shown some efficacy in eliminating GBM tumor cells, the inconsistency in therapeutic outcomes drives the search for new pharmacologic agents. Many of the identified pharmacologic agents do not reach therapeutic concentrations in the brain due to the highly impermeable BBB.^10^ Therefore, identifying and exploring effective drug delivery strategies that could circumvent the highly‐regulated BBB is critical in overcoming current limitations in both standard and experimental GBM treatment.

## 3 DRUG DELIVERY STRATEGIES THAT CIRCUMVENT THE BBB

### 3.1 Direct intracranial drug delivery into interstitial spaces of the brain

One way to bypass the BBB is by directly administering drugs into the interstitium of the brain, hence minimizing systemic side effects. An accepted approach is by encapsulation within a bioresorbable implant which is deposited into the resection cavity following tumor resection.^38^ To date, GLIADEL wafers, which facilitate controlled release of the alkylating agent carmustine, are the only FDA‐approved chemotherapeutic implants for GBM treatment.^39^ However, while they have led to increased OS in cohort studies, several randomized controlled trials have shown little improvement in survival outcomes, and instead reveal numerous toxicities in GBM patients.^39^ Moreover, these wafers mainly target tumor cells in close proximity to the site of implantation and have limited effect on disease which has infiltrated deep into the surrounding tissues. There have been recent improvements to interstitial delivery devices in the form of a biocompatible polymeric net that can be impregnated with chemotherapeutic drugs (MicroMESH). Unlike rigid interstitial wafers, this elastic, biodegradable microscopic net conforms to the shape of the remnant tumor bed, improving the homogeneity of drug delivery.^40^

Another technique for intracranial administration of drugs is convection‐enhanced delivery (CED), which utilizes a catheter inserted directly into the tumor to generate a pressure gradient that disseminates the drug to the target site. This enables delivery of substantial drug volumes and allows therapeutic concentrations to be achieved in the brain. However, as with GLIADEL wafers, they have limited efficacy in targeting infiltrative GBM tumor cells at distant sites. The invasive nature of this technique also decreases its attractiveness to both patients and clinicians. It is hence unsurprising that no drugs have yet been approved for use with CED despite multiple clinical trials since its inception in 1994.^41^

### 3.2 Magnetic resonance‐guided focused ultrasound (MRgFUS)

**FIGURE 2:**
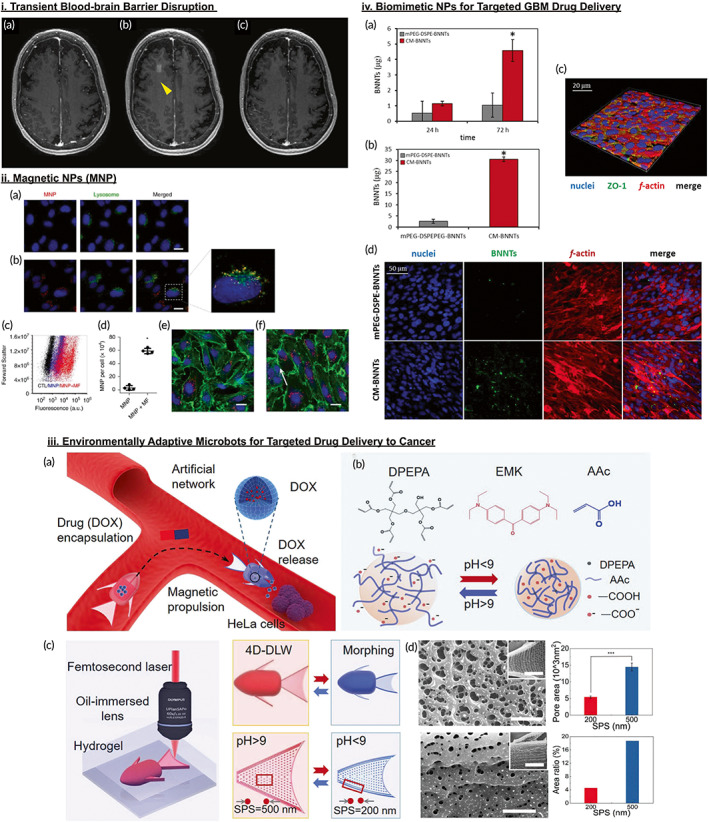
Collection of past research studies demonstrating the success of magnetic resonance‐guided focused ultrasound (MRgFUS), magnetic nanotechnology, and cancer cell membrane‐coated nanotechnology in cancer therapy. (i) Axial T1‐weighted gadolinium MRI of blood–brain barrier (BBB) opening and closure during MRgFUS therapy: (a) pre‐treatment, (b) immediately after sonication, with contrast extravasation observed in right frontal lobe due to BBB opening, and (c) 24 h after procedure, with no visible contrast extravasation due to BBB closure. (ii) Effect of magnetic field on uptake of Magenetic Nanoparticles (MNPs) by endothelial cells: (a) Increased uptake with magnetic field (b) Insignificant uptake without magnetic field. (c) Cellular uptake of MNPs with or without magnetic field analyzed using flow cytometry. (d) Quantitative analysis of MNPs within cells by measuring iron content. When endothelial cells containing MNPs were further incubated without (e) or with (f) a magnetic field, the stress fibers (green) of endothelial cells became aligned with the direction of the magnetic force in (f). (iii) Four‐dimensional (4D) printing of magnetic shape‐morphing microfish (SMMF) using pH‐sensitive hydrogels that could expand at pH >9 and contract at pH <9, allowing the release of doxorubicin (DOX) at low pH. (a) Schematic illustration of how SMMF can be used in the treatment of HeLa through controlled DOX release. (b) Chemical composition of hydrogels and changes that occur during SMMF expansion and contraction. (c) 4D printing of a SMMF that enables shape morphing. (d) Scanning electron microscopy images and quantitative analysis of the pores in SMMFs. (iv) CM‐BNNTs showing enhanced penetration across the in vitro BBB model compared to mPEG‐DSPE‐BNNTs (control) in (a) and (b). (c) Analysis of the endothelial cells of the BBB model using immunostaining. (d) Analysis of BNNT uptake by endothelial cells using confocal imaging (green). Figures were reproduced with permission from (i) in Ref. 44, (ii) Ref. 53, (iii) Ref. 54, (iv) Ref. 87. 4D‐DLW, 4D‐direct laser writing; AAc, acrylic acid; DPEPA, dipentaerythritol pentaacrylate; EMK, 4,4′‐bis(diethylamino)benzophenone; mPEG‐DSPE, 1,2‐distearoyl‐sn‐glycero‐3‐phosphoethanolamine‐N‐[methoxy(polyethylene glycol); SPS, scanning point spacing; ZO‐1, Zona occludens‐1/tight junction protein‐1

An alternative strategy being explored to overcome the impermeable BBB is by inducing a transient opening to grant passage for therapeutics delivery. MRgFUS is a noninvasive method that combines a low intensity FUS together with systemically injected microbubbles to mechanically disrupt the BBB without causing heat injury.^42^, ^43^ This technique has been demonstrated elegantly by Lipsman et al., who utilized serial gadolinium contrast‐enhanced MRIs to capture the temporary extravasation of contrast within a 24‐h window post‐sonication, indicating BBB disruption followed by reclosure (Figure 2i).^44^ In the same vein, a study examining the use of MRgFUS for pre‐resection chemotherapy in high‐grade glioma patients demonstrated higher chemotherapeutic drug concentrations in sonicated compared to unsonicated tissues.^45^ Furthermore, no adverse side effects as a result of the procedure were observed. Despite its small sample size, this study highlights the potential of MRgFUS in opening the BBB and enhancing drug delivery to the brain. Nevertheless, more statistically‐powered studies evaluating MRgFUS are required before it can be implemented in GBM patients. Studies centered on harnessing the extent and duration of BBB disruption are also paramount as an indefinite opening of the BBB will predispose the brain to infection or drug‐induced cerebral toxicity.^46^, ^47^

### 3.3 GBM‐targeted NPs

Over the years, many types of NPs have been developed to enhance permeability across the BBTB and concentrate drug delivery at target sites to minimize systemic toxicity.^48^ Different classes of NPs possess different physicochemical traits which confer each of them specific advantages. Lipid‐based NPs such as liposomes, solid lipid NPs and nanostructured lipid carriers efficiently penetrate the BBTB due to their lipophilic properties.^49^ On the other hand, polymeric NPs made from polylactic acid (PLA), polyglycolic acid (PGA), and polylactic‐co‐glycolic acid (PLGA) are known for their prolonged half‐life and superior drug release kinetics.^50^ Metallic NPs in particular, which belong to the category of inorganic NPs, are generated from different metal elements or composite magnetic metal oxides,^51^ and are gaining traction due to their potential for many applications including magnetic imaging and therapy.^52^ Magnetite nanocrystals coated with phospholipid‐PEG (polyethylene glycol) have been used to explore the effects of intracellular magnetic NPs on endothelial TJs in the presence of an external magnetic field. As demonstrated in Figure 2ii, when the magnetic field was applied, a large uptake of magnetic NPs by endothelial cells was observed. This corresponded to a change in the configuration of F‐actin fibers in endothelial cells, from being randomly distributed to uniformly aligned in the direction of the magnetic force.^53^ This resulted in temporary disruption of endothelial TJs followed by activation of the paracellular transport pathway, hence promoting movement of all circulating agents across the BBB.^53^ Recently, extraordinary advancements in magnetic technology, such as magnet‐guided fish‐shaped microrobots that could release chemotherapeutic drugs in response to a decreased pH, which simulates the acidic TME, have also been developed (Figure 2iii).^54^ If a magnetic field strong enough to penetrate the cranium and its contents to exert effects on the BBB could be developed, this novel technique could usher in a new paradigm of drug delivery to the brain.^55^

Despite the immense potential of these NPs in treating GBM, their efficacy remains limited by their susceptibility to clearance by the reticuloendothelial system (RES) due to the presence of numerous immunogenic molecules on their surface. This significantly decreases the number of NPs that eventually reach their target.^50^ While surface modification of NPs with zwitterionic ligands such as cysteine, glutathione, or PEG can reduce immunogenicity and prolong circulation time, no NP formulation to date can completely facilitate evasion of immune clearance.^56^ Therefore, development of NPs with lower immunogenicity and greater biocompatibility is crucial for improved NP delivery to the brain.

## 4 BIOMIMETIC NANOPARTICLES

As with most drugs, the efficacy of traditional NPs is limited by their relative inability to pass through the BBB, with research showing that only 0.7% of the administered NP dose is successfully delivered to tumors in the brain.^57^ Hence, novel NP designs that can bypass the BBB and specifically target GBM tumor cells are imperative for effective therapy. The integration of biomaterials mimicking characteristics of biological molecules and cells has been shown to increase NP uptake at the BBB through natural ligand‐receptor interactions.^58^ These biological mimics, also referred to as biomimetic NPs, are significantly less immunogenic and also possess greater specificity for GBM tumor cells expressing biomimetic complements.^59^ Table 1 highlights the advantages of using biomimetic NPs compared to traditional NPs, focusing on the increased stability, higher drug loading capacity and enhanced safety profile conferred by biomimetic NPs. Clinical trials have also been implemented for several NPs, but many require further work to fully elucidate its performance (Table 2).

**TABLE 1:** Comparison between biomimetic and traditional NPs, highlighting the merits of biomimetic NPs as drug delivery carriers for GBM.

	Lipid‐based NPs	Polymeric NPs	Inorganic and metallic NPs	Biomimetic NPs
Examples	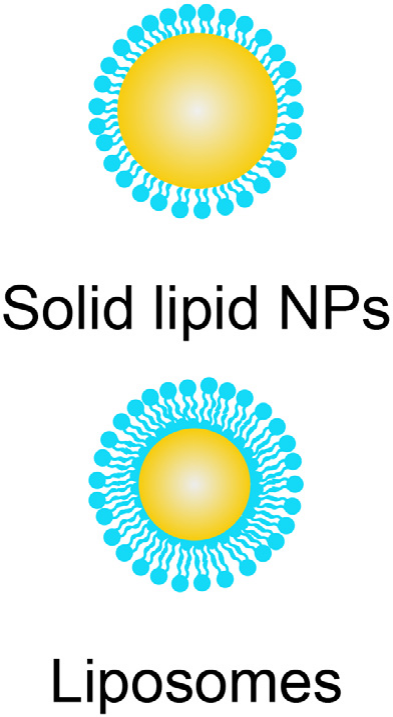	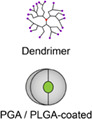	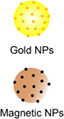	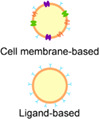
Advantages	Suitable carrier for both hydrophilic and hydrophobic drugs^133^ Easy to manufacture^133^	Easy surface modification^134^ Customizable to desired environment^135^	Higher targeting specificity with usage of external magnetic field^136^ Wide range of applications, including theranostics^137^	Low immunogenicity^58^ Versatility in surface coatings for increased target specificity^58^
Stability	Poor, due to oxidation and hydrolysis, but can be enhanced using PEG^138^	High, due to ability to form ionic and covalent bonds^139^	High, due to potential for stable bonds to be formed between NP core and drug^140^	High, due to surface coating optimization for transport in the body^141^
Encapsulation efficiency	Low, due to reasons such as limited space of lipid bilayer in liposomes^133^	Low, due to high molecular weight^133^	High, due to ability to modify surface chemistry of NPs^142^	High, due to wide variety of biomolecules that can accommodate various physicochemical properties of drugs^141^
Safety profile	Generally nontoxic as shown in several GBM clinical trials^143^, ^144^, ^145^, ^146^	Specific types of polymers e.g. PLGA, PGA, PLA are less toxic than other types,^50^ but no GBM clinical trial to date	Generally more toxic than the other NPs, but a clinical trial done on gold NPs showed no severe adverse side effects^147^	Low toxicity due to biomimicry,^148^ with a few successful GBM clinical trials done on viral vectors^149^, ^150^

Abbreviations: GBM, glioblastoma multiforme; NPs, nanoparticles; PGA, polyglycolic acid; PLA, polylactic acid; PLGA, polylactic‐co‐glycolic acid.

**TABLE 2:** Nanoparticles that have undergone clinical trials for GBM therapy.

Name	Nanoparticle type	Biomimetic conjugates (if any)	Drug delivered in NPs	Administration	Patients	Phase	Status	Outcome	Clinical Trial ID/Reference
DaunoXome	Liposomes	—	Daunorubicin	Only with DaunoXome	Recurrent or progressive brain tumors in children	—	Completed	High response rate, reducing tumor growth significantly	144
Caelyx	PEGylated liposomes	—	Doxorubicin	Administered with oral TMZ	Recurrent GBM	Phase II	Completed	Median PFS of 3.2 months. Median OS of 8.2 months	NCT00944801/145
NL CPT‐11	PEGylated liposomes	—	Irinotecan	Only with NL CPT‐11	Recurrent HGG	Phase II	Completed	Only 17.6% meet the desired outcome of PFS 6 months.	NCT00734682/146
AdV‐tk	Viral Vectors	Adenoviral vector	Gene transfer of HSV1‐TK	Administered with Valacyclovir (antiviral) and radiation therapy	HGG	Phase II	Completed	Outcome not listed but phase IB showed 25% 3‐year survival rate	NCT00589875/149
2B3‐101	PEGylated liposomes	Glutathione	Doxorubicin	Only with 2B3‐101	Recurrent HGG	Phase I	Completed	40% achieved PFS 3 months	NCT01386580/143
Myocet	Liposomes	—	Doxorubicin	Only with Myocet	Recurrent HGG in children	Phase I	Completed	Median PFS of 2 months	NCT02861222/151
Toca 511/Toca FC	Viral Vectors	Retroviral vectors	Gene transfer of cytosine deaminase	Administered with TMZ and undergoes radiation therapy	Newly diagnosed GBM	Phase II/III	Withdrawn	No significant difference in median OS in treatment arm vs control arm	NCT04105374/96
NU‐0129	Gold nanoparticles	siBcl2L12	—	Only with NU‐0129	Recurrent GBM	Early Phase I	Completed	NPs crossed BBB and accumulate in glioma cells	NCT03020017/147
Onyvide	PEGylated liposomes	—	Irinotecan	Administered with Metronomic TMZ	Recurrent GBM	Phase 1	Terminated	Limited responses; median PFS of 2 months	NCT03119064/152
C225‐ILs‐Dox	DSPE‐PEGylated liposomes	Anti‐EGFR mAb (Cetuximab)	Doxorubicin	Only with C225‐ILs‐Dox	EGFR+ GBM	Phase I	Completed	Median PFS of 1.5 months. Median OS of 8 months.	NCT03603379/153
Ad‐hCMV‐TK and Ad‐hCMV‐Flt3L	Viral vectors	Adenoviral vectors	Gene transfer of HSV1‐TK and Flt3L	Administered with Valacyclovir (antiviral)	HGG	Phase I	Completed	Median PFS of 9.9 months. Median OS of 21.3 months.	NCT01811992/150
SGT‐53	DOTAP:DOPE cationic liposome	Anti‐TfR mAb	Plasmid encoding WT p53 cDNA	Administered with oral TMZ	Recurrent GBM	Phase II	Terminated	Outcome not listed.	NCT02340156

Abbreviations: Ad, adenovirus; Flt3L, Fms‐like tyrosine kinase 3 ligand; HGG, high‐grade glioma; HSV1‐TK, herpes simplex virus type 1‐thymidine kinase.

As illustrated in Figure 1b–d, biomimetic NPs are taken up by GBM tumor cells through a variety of methods. Absorptive transcytosis involves electrostatic forces of attraction for enhanced BBB penetration by biomimetic NPs. Endothelial cells in the BBB are made up of phospholipid‐rich cell membranes coated with a glycocalyx and other carboxyl groups of sialoglycoproteins and sialoglycolipids, making them negatively‐charged.^60^ Cationic ligands attached to the surface of NPs confer positive charges, thus facilitating absorptive transcytosis across the BBB via electrostatic forces.^61^ Another unique method for drug delivery to GBM tumor cells involves receptor‐mediated transcytosis, a highly selective and specific process relying on receptors present on the surface of both endothelial cells in the BBB and GBM tumor cells.^8^ Last but not least, macropinocytosis is a phenomenon involving the remodeling of actin cytoskeletons associated with the plasma membrane. This results in a change in the configuration of the membrane with an extension of the cell curving inwards, eventually engulfing extracellular contents such as NPs and extracellular fluid.^62^, ^63^

In this review, biomimetic NPs are classified into four major categories—protein‐based, ligand‐based, cell membrane‐based, and natural transporter‐based types, all of which present unique solutions to the limitations of traditional NPs.

### 4.1 Protein‐based biomimetic NPs

Many serum proteins circulating in our bloodstream have been explored as possible drug delivery carriers for GBM treatment. Albumin, the most abundant protein in plasma, has been extensively explored for biomimetic NP design. Its long circulatory half‐life enables albumin‐based NPs to remain in circulation for a longer period of time and accumulate in tumors at higher concentrations.^64^ Albumin also interacts with receptors such as secreted protein acidic and rich in cysteine (SPARC) and glycoprotein 60 (gp60), which are overexpressed in endothelial cells undergoing increased neovascularisation as well as GBM tumor cells, thus allowing a larger number of albumin‐based NPs to infiltrate the tumor.^58^, ^65^, ^66^

Human H‐ferritin (HFn) NPs, designed by Fan et al., were able to traverse the BBTB and accumulate within GBM tumor cells, while sparing surrounding normal brain tissue.^67^ The high expression of human HFn receptors in both BBB endothelial cells and GBM cells facilitates receptor‐mediated transcytosis of HFn NPs carrying therapeutic payloads.^68^ This was evident in U87‐MG bearing mice where doxorubicin (DOX)‐containing HFn NPs resulted in a visible regression of tumor growth and prolonged survival time compared to those treated with free DOX. The concept of mimicking natural proteins in our body opens many doors for the future development of NPs.

### 4.2 Ligand‐based biomimetic NPs

Biomimetic NPs that are coated with natural GBM‐specific ligands such as folate and lactoferrin have been developed in many studies. Folate, also known as vitamin B9, is a commonly used ligand to increase tumor specificity of biomimetic NPs.^69^ This is because folate receptors, specifically the FRα subtype, are overexpressed in many tumors including GBM and tumor‐associated blood vessels, as folate is crucial in nucleotide synthesis and hence tumor expansion.^70^ Folate‐conjugated NPs are readily endocytosed by GBM cells and broken down to release their encapsulated drugs.^71^ In a study utilizing folate ligands to functionalise TMZ‐coated magnetite NPs, conjugated NPs produced a 2.37‐fold higher cytotoxicity in C6 cancer cells compared to nonconjugated NPs,^72^ reflecting folate's potential as a ligand to confer GBM‐targeting capabilities to many chemotherapeutic drugs. Lactoferrin is another known brain‐targeting ligand that easily crosses the BBB through receptor‐mediated endocytosis.^73^ PEG‐coated bovine serum albumin (BSA) NPs coated with lactoferrin have been designed to deliver DOX in vivo in glioma‐bearing rats.^74^ These NPs resulted in higher accumulation and increased cytotoxicity in the tumor compared to nonlactoferrin coated NPs.

Monoclonal antibodies (mAb) specific for receptors found on GBM tumor cells can also be synthesized to increase homing capacity of NPs. An antitransferrin receptor mAb has been designed and used to coat poly(β‐l‐malic acid) NPs to target endothelial cells in the BBB which overexpress transferrin receptors, allowing these NPs to cross the BBB with greater ease. An additional anti‐EGFR mAb (cetuximab) was also added to enable these NPs to target GBM tumor cells with increased EGFR expression.^75^

In addition, short peptides derived from naturally occurring proteins can be used as ligands to enhance specificity of biomimetic NPs for drug delivery. Low‐density lipoprotein receptors (LDLRs) are often overexpressed in GBM and are responsible for the movement of many molecules across the BBB. An apolipoprotein B derived peptide, ApoB29 mer peptide, is a ligand that binds with high affinity to LDLRs and was used in a study by Seo et al.^76^ The study showed that ApoB29‐conjugated gold NPs penetrated the BBB more efficiently compared to bare gold NPs and preferentially accumulated in the tumor microenvironment (TME), demonstrating the potential of ApoB29 mer peptide as a ligand to increase NP specificity for GBM tumor cells.

In another study, albumin‐based NPs were conjugated with a short peptide that can bind to transferrin receptors on the BBB and GBM tumor cells.^77^ This short peptide, transferrin receptor (TfR)‐binding peptide T12, binds to a different part of the receptor away from the site of endogenous transferrin binding, avoiding competitive inhibition of the naturally occurring protein. The NPs were also embellished with an additional sugar moiety, mannose, which bound to mannose receptors on protumor M2 tumor‐associated macrophages (TAM2). The codelivery of disulfiram/copper complex and regorafenib resulted in an efficient inhibition of GBM cell proliferation and also successfully programmed TAM2 towards the antitumor M1 (TAM1) phenotype, enhancing the antitumor immune response. This shows how ligands specific for GBM cells and cells in the TME can enhance localization of NPs for increased antitumoral effects.

Many other ligands that have been used to synthesize biomimetic NPs belong to a novel category called cell penetrating peptides (CPPs). CPPs are made up of short polypeptides of about 5–30 amino acids. The iRGD peptide is an example of a CPP which binds to integrins and neuropilin‐1 receptors to allow endocytosis of substances into cells. It is often used for targeting angiogenic endothelial cells in GBM. In a study conducted by Liu et al., NPs with iRGD CPP demonstrated increased uptake and were able to selectively accumulate in GBM cells at higher concentrations in preclinical experiments.^78^ These examples serve to illustrate the wide array of ligands that can be utilized to increase NP penetration across the BBTB, giving rise to many possible permutations for future development.

### 4.3 Cell membrane‐based biomimetic NPs

The key advantage of NPs coated with natural cell membranes is their hypoimmunogenic physicochemical properties as they are able to evade immune surveillance by the RES, thereby increasing their time in circulation.^79^ To achieve this, various source cells including anucleate cells, prokaryotes, and eukaryotes, can be used to tailor‐make biomimetic NPs to suit a variety of purposes.^80^ Cell membranes from the cells‐of‐interest are first isolated under hypotonic conditions after homogenization and centrifugation, before being coated onto NPs via a sonication/extrusion process.^81^ Biomimetic cell membrane‐coated NPs possess the antigenic diversity of the source cells as well as their functions, including immune camouflage, longer circulation time, and tumor‐specific targeting.^82^ In the context of GBM, NPs coated with immune cell membranes are of special interest due to the naturally antagonistic interactions between GBM cells and immune cells such as macrophages, natural killer (NK) cells, microglia, and T lymphocytes.^83^ Clinically, biopsy samples can also be expanded indefinitely in vitro to generate sufficient cell membrane for NP coating, making therapeutics centered on this technology more than viable.

#### 4.3.1 Immune cell membrane‐coated biomimetic NPs

Among the various types of immune cells, macrophage‐coated NPs have been used in several experimental studies for GBM therapy for their exceptional ability in traversing the BBTB. Macrophage‐coated near‐infrared Ib (NIR‐Ib) fluorescent dye IR‐792 NPs (MDINPs) used with NIR‐Ib imaging‐guided photothermal therapy in an orthotopic mouse model has shown heartening results in treating GBM.^84^ In this study, increased MDINP penetration across the BBTB was achieved through the binding of macrophage‐associated membrane proteins (e.g., integrin α4 and mac‐1) to endothelial cell receptors such as VCAM‐1 and ICAM‐1, which facilitated the transmigration of the NPs across the BBTB just like how they normally function as adherence and migration points for immune cells.^80^

In addition, NK cell membranes have also been used as coating substances for biomimetic NPs in GBM therapy. NK cell nanorobots with aggregation‐induced emission (AIE) characteristics (NK@AIEdots) have been developed by coating NK cell membranes on an AIE‐active conjugated polymer.^85^ The binding of NK@AIEdots via lymphocyte function‐associated antigen 1 (LFA‐1) and very late antigen‐4 (VLA‐4) with cell adhesion molecules on the BBB endothelial cells was found to trigger an intracellular signaling cascade, which disrupted TJs and reorganized actin cytoskeletons to form intercellular gaps at the BBB. Furthermore, the NK@AIEdots demonstrated increased uptake by GBM cells through the receptors, DNAX accessory molecule (DNAM‐1) and natural killer Group 2D (NKG2D), on the NK cell membrane, significantly inhibiting tumor growth under near‐infrared light illumination.^80^ This shows the potential of NK cell membranes in helping NPs traverse the BBTB and achieve better therapeutic outcomes.

#### 4.3.2 Cancer cell membrane‐coated biomimetic NPs

Cancer cells have also been explored as potential sources of cell membranes to synthesize membrane‐coated NPs. The tumor specific proteins used to coat these NPs include cell adhesion molecules such as cadherins, selectins, and integrins, which enable homologous binding to the source cell line to bestow a potent homing effect.^86^ De Pasquale et al. utilized boron nitride nanotubes (BNNTs) loaded with DOX and coated with cell membranes extracted from GBM cells (CM‐BNNTs) to demonstrate better BBTB penetration and uptake into the endothelial cells compared to the control (mPEG‐DSPE‐BNNT), as seen in Figure 2iv. Through pH‐dependent release of DOX, the CM‐BNNTS could then specifically target and kill GBM cells.^87^ Cell membranes extracted from U87MG brain tumor cells have also been used to coat lanthanide‐droped NPs (CC‐LnNPs) in another study.^88^ Compared to LnNPs, CC‐LnNPs showed significantly decreased phagocytic uptake and increased internalization by U87MG cells, demonstrating a tenfold higher fluorescence intensity after the NPs were incubated with the cells for 2 h. Primary cancer cells can therefore contribute to the range of cell membrane sources used to synthesize biomimetic NPs.

### 4.4 Natural transporter‐based biomimetic NPs

Naturally occurring transporters in the body such as high‐density lipoprotein (HDL), low‐density lipoprotein (LDL) and exosomes can be used as the base for biomimetic NP design. HDLs, in particular, are a popular choice for nanocarriers, as they are able to remain in circulation for an extended period of time, allowing accumulation of NPs at tumor sites. Similar to natural cell membranes used to coat biomimetic NPs, HDLs already possess in‐built ligands such as apolipoprotein A1 which recognize HDL scavenger receptors in the BBB and on GBM tumor cells.^89^ HDLs are small particles involved in reverse cholesterol transport,^64^ and are ideal for drug delivery due to their small size and nanodisc configuration which enables them to diffuse through dense tumors and accumulate at high concentrations.^12^ Synthetic HDL nanodiscs loaded with cytosine‐phosphate‐guanine (CpG), a toll‐like receptor 9 (TLR9) agonist, were formulated to deliver docetaxel to the GBM TME in GBM‐bearing mice in vivo.^65^ The HDL nanodiscs solely accumulated in the tumor with minimal amounts found in other organs, thus demonstrating the homing ability of these nanodiscs. Besides the cytotoxic effect of docetaxel, tumor cell death was also triggered by CpG which promoted the engulfment of tumor antigens by antigen presenting cells in the TME, hence promoting antitumoral CD8^+^ T cell‐mediated immunity and enabling the development of long‐term immunological memory against subsequent tumor antigens from recurrent GBM.

Low‐density lipoproteins are structurally similar to that of HDLs and have apolipoproteins that are recognized by in vis found on endothelial cells lining the BBTB and GBM tumor cells. In a study, synthetic nano‐LDL particles were synthesized by combining a peptide containing a lipid binding motif and the LDLR binding domain of apolipoprotein B‐100 with a lipid emulsion.^90^ The synthetic nano‐LDLs demonstrated high specificity for the LDLRs in glioma cell lines and can be used as drug delivery carriers for increased targeting of GBM tumor cells.

Exosomes, a type of extracellular vesicle, are naturally occurring transporters responsible for the intercellular transfer of molecules.^91^ Like HDLs, they are suitable nanocarriers due to their compact size and longer circulation time. Exosomes can be engineered to deliver desired drugs to GBM tumor cells. In one study, microRNA‐21 sponge construct was packaged into specially designed exosomes for delivery to GBM tumor cells, resulting in the downregulation of microRNA‐21 expression in the glioma cell lines U87MG and C6. Remarkably, when the same experiment was conducted in a GBM‐bearing rat model, there was a significant reduction in tumor size,^92^ highlighting the potential of exosomes as drug delivery carriers.

Other natural transporters that have been explored for drug delivery to GBM tumor cells are viruses. Viral vectors have long been used as a delivery platform for gene therapy. Retrovirus (RV), adenovirus (ADV), adeno‐associated virus (AADV), and herpes simplex virus‐1 (HSV) are the most commonly used viral vectors for GBM gene therapy.^93^, ^94^ Viral vectors are highly advantageous due to their ability to carry a large amount of genetic material and evade immune surveillance. Many viruses, especially retroviruses, are also able to maintain gene expression for a prolonged period of time due to the presence of reverse transcriptase which transcribes genetic material from single‐stranded RNA into double‐stranded DNA for integration into the host cell's genome.^95^ A phase II/III randomized clinical trial on vocimagene amiretrorepvec, a retroviral replicating vector carrying a transgene for cytosine deaminase which converts 5‐fluorocytosine into 5‐fluorouracil (5‐FU), was conducted in patients with recurrent GBM and anaplastic astrocytoma.^96^ 5‐FU is an antimetabolite that inhibits thymidylate synthase, suppressing DNA replication and RNA synthesis.^97^ However, the findings did not show any significant difference in OS between the treatment and control groups. Another phase III clinical trial using retroviral vectors with HSV thymidine kinase (HSV‐Tk) gene and ganciclovir for GBM gene therapy was performed where HSV‐Tk acts as a suicide gene and converts ganciclovir to its active form, halting cell cycle progression and eventually leading to cell apoptosis.^98^ Like many other viral vector gene therapies in GBM, the retroviral vector with HSV‐Tk showed limited improvement in patients' time to tumor progression and OS, which can be attributed to the failure of the viral vectors in delivering the drugs to the target tumor cells. This demonstrates a clear need for other methods to complement viral vectors as drug delivery carriers.

Recent developments in the field have given rise to viral‐mimetics that can be conjugated with NPs to improve homing capacity to the CNS. Gold‐liposome NPs conjugated with brain targeting peptides—rabies virus glycoprotein (RVG), and apolipoprotein E, were designed to improve the delivery of RNA interference (RNAi) for GBM treatment.^99^ RVG is a neurotropic viral peptide which binds to nicotinic acetylcholine receptors expressed in the BBB endothelium, neurons and GBM tumor cells. These NPs demonstrated increased delivery of RNAi to GBM cells compared to liposomes without the RVG peptide, and was able to downregulate the expression of miR‐92b, the target of RNAi. This shows how the combination of natural transporters and NPs can work synergistically to enhance drug delivery to GBM tumor cells.

The numerous examples listed above have highlighted improved antitumoral outcomes with biomimetic NPs when compared to traditional NPs. However, despite all these promising developments in the field of biomimetic NPs, translation from preclinical to clinical setting remains a work in progress. Henceforth, more effective targeting strategies that augment current biomimetic NPs are imperative.

## 5 STRATEGIES TO FURTHER ENHANCE NP‐MEDIATED GBM TUMOR KILLING

As mentioned previously, efficacy of most drugs including NPs, is often reduced due to inadequate BBTB penetration and the tricky characteristics of solid tumors, especially tumor heterogeneity. Current GBM treatment is also limited by the immunosuppressive TME, irregular vasculature causing an uneven distribution of systemically administered drugs,^100^ as well as the natural clumping of tumor cells restricting NP access.^101^ The following strategies are aimed at overcoming some of these limitations.

### 5.1 Combinatorial antigen targeting with CAR‐T cells and NPs

Tumor specificity is a key, yet often limiting factor in the efficacy of biomimetic NPs, especially in a highly heterogeneous tumor like GBM. One method to resolve this issue is the use of chimeric antigen receptors (CARs). CARs consist of an extracellular antigen‐specific domain comprising monoclonal antibody fragments that is fused to an intracellular domain made up of the CD3ζ chain of the TCR and costimulatory molecules for signaling purposes.^102^ CARs that are specific for GBM tumor antigens can be artificially expressed on genetically modified T cells collected from patients' peripheral blood.^103^ Interleukin‐13 receptor alpha 2 (IL13Rα2), epidermal growth factor receptor variant III, human epidermal growth factor receptor 2 (HER2) and erythropoietin‐producing hepatocellular carcinoma A2 (EphA2) are some examples of GBM‐specific antigens that have been employed in CAR design.^104^ Other newly developed targets include ganglioside 2, B7‐H3, chlorotoxin, and CD70.^104^, ^105^

These molecules have all been clinically verified as safe targets of CAR‐T therapy for GBM, but only two CAR‐T therapies specific to CD19+ B cell malignancies are currently approved by the FDA.^106^ CAR‐T therapies for GBM are still mostly in phase I clinical trials, where the safety and efficacy of second‐generation CAR‐T cells are still being evaluated.^107^ Unfortunately, an insignificant antitumor response has been reported in clinical trials. This can be attributed to the dense extracellular matrix (ECM) and immunosuppressive TME comprising regulatory T cells and immune checkpoint molecules which severely restrict the trafficking and expansion of systemically administered CAR‐T cells at the tumor site.^108^ This makes it difficult for T cells to infiltrate and persist in solid tumors.^108^

**FIGURE 3:**
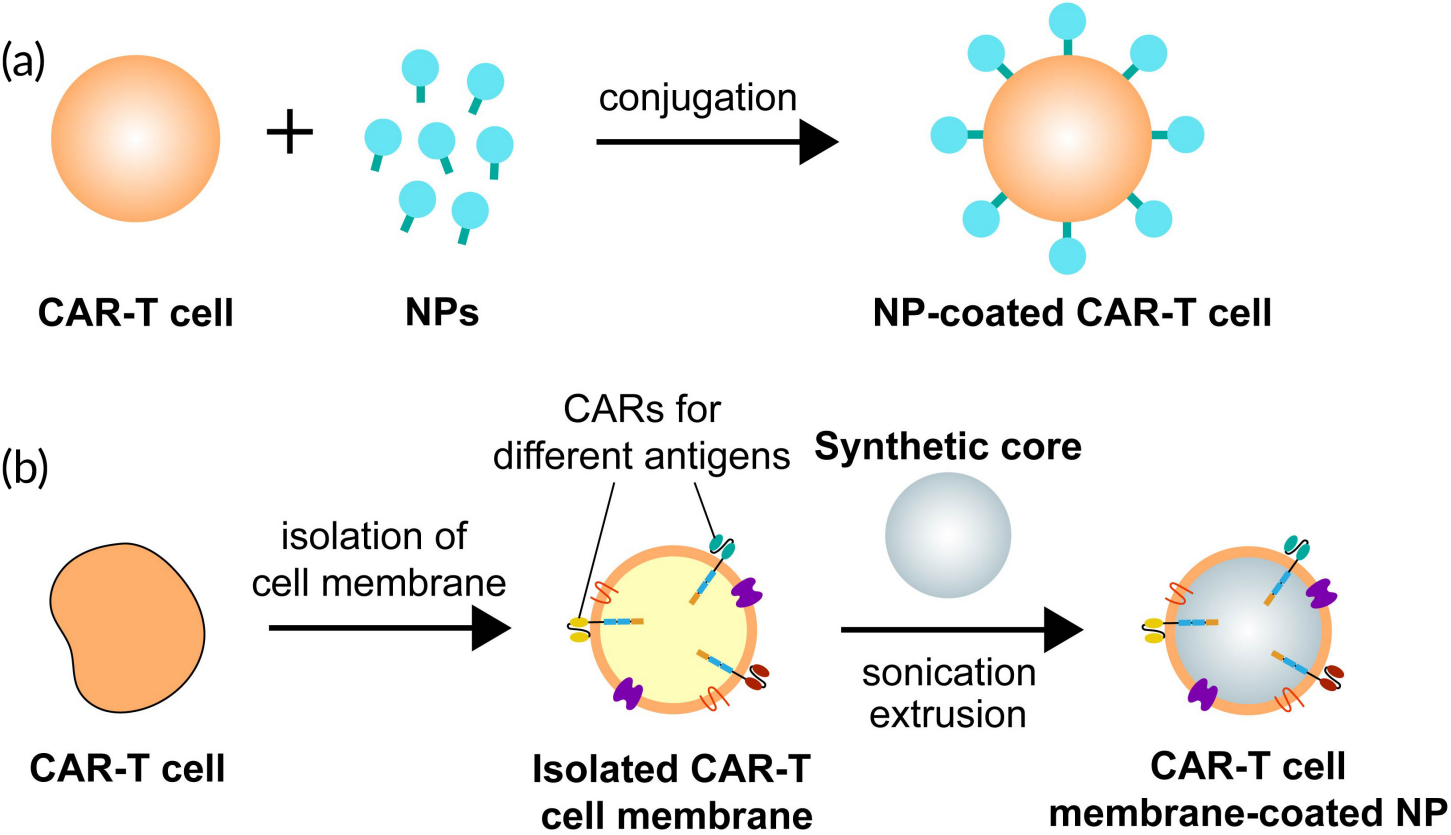
Strategies to enhance glioblastoma multiforme (GBM) tumor killing by nanoparticles (NPs). (a) Conjugation of chemotherapeutic drug‐loaded NPs onto the surface of chimeric antigen receptor (CAR)‐T cells to increase delivery of NPs to GBM tumor cells. (b) CAR‐T cell membrane‐coated NPs with multiple CARs targeting a variety of antigens on tumor cells.

That being said, the combination of CAR‐T cell technology and biomimetic NPs to create a new therapeutic vehicle could potentially overcome the immunosuppressive TME in GBM. As a single CAR‐T cell membrane can be modified to carry a tailored blend of tumor specific antigens, they are especially useful in targeting heterogeneous tumors like GBM.^109^ A single universal (U) tricistronic transgene (UCAR) has successfully generated CAR‐T cells that simultaneously expressed multiple CAR molecules specific for HER2, IL13Rα2, and EphA2, which were able to capture nearly 100% of GBM tumor cells. This creates much optimism in combinatorial antigen targeting as a potential strategy to overcome the antigenic heterogeneity found in GBM.^110^ This is especially so when conjugated with biomimetic NPs, facilitating the creation of a new therapeutic vehicle that can achieve effective tumor killing without having to rely on CAR‐T cell action (Figure 3a). In a study by Kim et al., CAR‐T cells were modified with a targeted‐quadruple‐mutant of IL13 (TQM‐13) and coated with DOX‐loaded NPs.^102^ TQM‐13 was shown to have high affinity for IL13Rα2‐expressing GBM cells with little off‐target effects. When empowered by a NP coating, these T cells displayed higher cytotoxic effects in vitro compared to bare T cells, and also achieved higher concentrations within the tumor in vitro and in vivo compared to NPs alone, demonstrating the synergistic efficacy of the NPs' high therapeutic payload capacity with the homing prowess of CAR‐T cells. In other areas of study, researchers have also used mesoporous silica containing IR780 NPs with CAR‐T cell membranes to specifically target GPC3‐expressing hepatocellular carcinoma (HCC) cells (Figure 3b). These NPs were more effective in targeting HCC cells compared to IR780‐loaded mesoporous silica both in vivo and in vitro, illustrating the ability of CAR‐T cell membranes to confer their high homing potential for tumor antigens through this method.^111^ These examples show how a combination of CAR‐T therapy and biomimetic NPs can enhance tumor killing by exploiting the benefits and compensating for the weaknesses of both technologies.

### 5.2 Biomimetic NPs with homologous targeting and TME‐responsive elements

Besides CAR‐T cell membranes, GBM tumor cell membranes can also be used for homologous targeting of NPs to GBM. Additional TME‐responsive elements can then be used to further increase the efficacy of tumor killing. Recently, a team of researchers formulated GBM cell membrane‐coated NPs, which demonstrated superior BBB penetration and GBM targeting through homotypic recognition, in comparison to bare NPs. These NPs contain lactate oxidase which convert lactic acid present in high concentrations in the TME into pyruvic acid and hydrogen peroxide. Pyruvic acid suppresses tumor cell growth while hydrogen peroxide reacts with the delivered bis[2,4,5‐trichloro‐6‐(pentyloxycarbonyl)phenyl] oxalate to release energy, which is subsequently utilized by the photosensitiser chlorin e6 for the generation of singlet oxygen to eliminate GBM cells.^112^ This underscores how homologous targeting increases the specificity of biomimetic NPs and can further enhance therapeutic outcomes by utilizing TME‐responsive substances to generate cytotoxic particles.

Another example would be the creation of GBM cell membrane‐coated NPs enveloping a pH degradable acetal grated dextran inner core loaded with TMZ and cisplatin.^113^ Under physiological conditions (pH 7.4), less than 20% of the drugs was released from the NPs due to their stable configuration. However, at pH 6.5 and pH 5.0 which simulate the acidic TME of GBM tissue, the percentage of drug release increased to 54% and 76% respectively within 24 h. This allows for controlled drug release at GBM cells and reduces the risk of off‐target cytotoxicity. The synergistic combination of homologous targeting and TME‐sensitive elements is hence able to concentrate drug delivery at the target tissues and enhance GBM tumor killing.

### 5.3 Aptamers to complement biomimetic NPs

Another strategy to increase target specificity of biomimetic NPs is the usage of aptamers, which are single‐stranded oligonucleotides that possess different configurations and bind to target molecules such as membrane‐bound proteins.^114^ These ligand‐receptor interactions lead to internalization of the aptamer‐containing molecule.^115^ This phenomenon has been beautifully demonstrated by Liu and his team, who designed DOX‐loaded, pH‐sensitive PLGA NPs coated with aptamer SL1‐modified red blood cell membranes (RNPs).^116^ SL1 binds specifically to c‐Met, a protein found at high concentrations in GBM U87MG cells, thus enabling the accumulation of DOX in the tumor cells. Unsurprisingly, the results revealed a higher uptake of SL1‐RNP‐DOX and an accelerated rate of tumor killing compared to GBM cells treated with unmodified RNP‐DOX, resulting in a prolonged median survival time. This provides great hope for the use of aptamers to complement existing biomimetic NP design for enhanced GBM specificity.

### 5.4 Enhancing biomimetic NP uptake through macropinocytosis

A large proportion of current research aims to enhance specificity of drug‐delivery carriers for the BBTB and GBM tumor cells. However, studies on the mechanisms to increase drug uptake from the interstitial space into tumor cells are far and few between. As mentioned earlier, macropinocytosis is a form of endocytosis that allows the nonselective uptake of proteins and extracellular fluids into cells via vesicles called macropinosomes.^117^ This process can be initiated by oncogenes or the activation of growth factor signaling pathways and represents a unique way for tumor cells to survive in the hostile TME through the uptake of nutrients such as amino acids.^118^ Macropinocytosis can be exploited for anticancer therapies either through direct suppression of the pathway or utilizing it to increase cytotoxic drug delivery to tumor cells. Apolipoprotein E3 (ApoE3)‐reconstituted HDL was used as a NP coating to encapsulate a siRNA‐loaded calcium phosphate core (CaP‐rHDL).^119^ The protein‐based NP coating demonstrated increased penetration across the BBTB for two reasons. First, the abundance of ApoE3 receptors in the BBTB and GBM cells (LDLR and LDLR‐related protein 1) facilitated NP homing to the tumor.^120^, ^121^ Second, the protein‐based carrier mimicked natural proteins recognized by GBM tumor cells, resulting in the large uptake following macropinocytosis activation. These NPs significantly prolonged the survival of the treated mice and highlight the big role macropinocytosis plays in increasing NP uptake by GBM tumor cells.

### 5.5 ECM‐digesting enzyme‐coated biomimetic NPs

After extravasating from blood capillaries in the BBB, NPs enter an interstitial space containing interstitial fluid that bathes the parenchymal cells of the brain and spinal cord.^122^, ^123^ They will then have to diffuse through an ECM comprising proteoglycans (PGs), glycosaminoglycans (GAGs) such as hyaluronic acid, and other glycoproteins,^124^ before being absorbed by tumor cells to exert their therapeutic effects. During cancer development, there is upregulated production of the ECM as well as fibro‐adhesive components such as type IV collagen, fibronectins and laminins,^125^ resulting in increased viscosity and density of the interstitium. An elevated interstitial fluid pressure (IFP) further restricts the passage of therapeutic agents through the interstitium.^126^ These conditions are associated with stromal thickening and lead to an irregular distribution of NPs throughout the tumor, impairing subsequent endocytosis‐mediated uptake of NPs into GBM cells.^127^

Besides capitalizing on ligand‐receptor interactions to improve NP uptake into GBM cells, the transport of NPs through the interstitial space can also be enhanced by breaking down the dense ECM using ECM‐digesting enzymes such as collagenase and hyaluronidase.^128^ In a study on GBM‐bearing mice, human adipose‐derived mesenchymal stem cells (MSCs) were loaded with an adenovirus expressing hyaluronidase (ICOVIR17) and encapsulated in biocompatible synthetic ECM.^129^ These stem cells loaded with ICOVIR17 resulted in a significant decrease in GBM tumor regrowth and increased mice survival. This study depicted how hyaluronidase can be used to mediate hyaluronic acid degradation and decrease ECM density in GBM. Similarly, hyaluronic acid‐coated micelles loaded with the chemotherapeutic agents gemcitabine and honokiol demonstrated increased uptake by GBM cells via CD44 receptor‐mediated endocytosis in another study.^130^ This resulted in a significantly increased suppression of tumor growth and longer survival rates of mice bearing orthotopic xenograft GBM.

Human recombinant hyaluronidase has also been successfully added to red blood cell membrane‐coated NPs and this conjugation was observed to enhance NP diffusion in the hyaluronic acid‐rich matrix surrounding PC3 prostate cancer cells.^131^ This can potentially be applied to GBM as well to increase NP distribution. This is especially true given that GBM is a highly proliferative tumor with a florid stroma and fibrous matrix. The incorporation of ECM‐digesting enzymes into biomimetic NP formulation is hence likely to enhance drug distribution within the tumor core.

### 5.6 Robust drug delivery method

The combination of biomimetic NPs with other specific types of therapy can also selectively enhance different therapeutic effects. PLGA nanovectors encapsulating silver NPs were coated with chlorotoxin (CTX) to target matrix metalloproteinase‐2 (MMP‐2) and chloride channel‐3 (CIC‐3), both of which are highly expressed on GBM cells.^132^ When these NPs were used in conjunction with radiation therapy, it was associated with improved targeting of scattered GBM cells as ionizing radiation damages the integrity of the BBB and induces MMP‐2 expression.^132^ This synergistic combination resulted in significantly delayed residual and metastatic tumor growth vis‐à‐vis monotherapy with biomimetic NPs alone, and shows how various strategies can be simultaneously employed for their synergistic effects to overcome limitations of GBM monotherapy.

## 6 CONCLUSION

Despite significant progress in the treatment of other cancers over the years, current treatment strategies for GBM often fall short of treatment goals. The current array of therapeutic tools remains inadequate, and is often blunted by acquired drug resistance by rapidly evolving GBM cells. To effectively pioneer a new paradigm of GBM treatment, overcoming the major obstacles faced today is paramount. One of which is the impermeable BBB, which precludes the penetration of several therapeutic drugs into the CNS. The need for improved drug delivery methods across the BBB so that better therapeutic strategies can be devised is clear, and biomimetic NPs are an extremely attractive proposition, especially if combined with other strategies such as CAR‐T technology and ECM‐digesting enzymes. The increased tumor specificity and decreased immunogenicity conferred by these particles could usher in a new age of improved therapeutic outcomes. Although further studies and enhancements are necessary to optimize these NPs for clinical use, nanotechnology is undoubtedly a field that possesses a wealth of untapped potential that could radicalize the treatment of GBM.

## AUTHOR CONTRIBUTIONS

**Xin Yuan Lim:** Conceptualization (equal); writing – original draft (equal); writing – review and editing (equal). **Sharah Mae Capinpin:** Writing – original draft (equal); writing – review and editing (equal). **Nagarjun Bolem:** Supervision (supporting); writing – original draft (supporting); writing – review and editing (supporting). **Aaron Song Chuan Foo:** Supervision (supporting); writing – original draft (supporting); writing – review and editing (supporting). **Wai‐Cheong George Yip:** Supervision (supporting); writing – original draft (supporting); writing – review and editing (supporting). **Daniel Boon Loong Teh:** Conceptualization (lead); writing – original draft (lead); writing – review and editing (lead).

## CONFLICT OF INTEREST

The authors declare no conflicts of interest.

### PEER REVIEW

The peer review history for this article is available at https://publons.com/publon/10.1002/btm2.10483.

## Data Availability

All data that supports this review are available upon reasonable request from the co‐corresponding authors.
